# Gorham-Stout disease successfully treated with sirolimus (rapamycin): a case report and review of the literature

**DOI:** 10.1186/s12891-020-03540-7

**Published:** 2020-08-25

**Authors:** Yu Liang, Ruicheng Tian, Jing Wang, Yuhua Shan, Hongxiang Gao, Chenjie Xie, Jingjing Li, Min Xu, Song Gu

**Affiliations:** Department of Surgery, Shanghai Children’s Medical Center, Shanghai Jiaotong University School of Medicine, Dongfang Road No.1678, Pudong District, Shanghai, 200127 China

**Keywords:** Gorham-Stout disease, Osteolytic lesion, Sirolimus (rapamycin), Treatment

## Abstract

**Background:**

Gorham-Stout disease (GSD) is a rare disease characterized by bone lesions and osteolysis. Therapy usually involves surgical resection. Sirolimus (Rapamycin) is used in some patients with GSD but the efficacy and safety of Sirolimus remains unclear. We propose that Sirolimus may be a novel therapeutic for GSD and present a case and review of literature that supports this.

**Case presentation:**

We presented a 1-year-old boy with GSD involving osteolysis of the right humerus with fracture of the left femur complicated by an effusion in the right pleural cavity. X-rays showed osteolysis in the right clavicle. A large pleural effusion was observed on the right-side, and the left lung was significantly compressed. X-rays also showed a fracture of the left femur. A femoral biopsy was performed that showed necrotic tissue in the cortical bone and a large number of irregularly shaped capillaries that proliferated within the necrotic tissue. Dilated lymphatic vessels were seen adjacent to the cortex, with fibrous tissue hyperplasia. We prescribed sirolimus, which is an oral mTOR inhibitor, for two consecutive years. The boy recovered well without other progressive bone lesions and participates in normal daily activities. His growth and development are the same as that of his peers.

**Discussion and conclusion:**

Gorham-Stout disease is a rare and enigmatic disease characterized by the presentation of an intraosseous lymphatic anomaly (LM), which results in progressive bone resorption. Based on this case report and a literature review, we conclude that sirolimus may be an effective alternative medication for GSD.

## Background

Gorham-Stout Syndrome, also known as GSD, results in massive osteolysis and bone loss. The disease was first reported in 1838 [1] but was systematically described in 1955 by Gorham and Stout based on their clinical experience and the literature [2]. To date, about 300 cases have been reported worldwide. Commonly, GSD is defined as progressive bone resorption, which occurs either slowly or rapidly, in association with proliferation of lymphatics or blood vessels in areas adjacent to the osteolytic bone. The cause of the abnormal proliferation of vascular and lymphatic channels remains unknown, although there are different hypotheses about pathogenesis involving vascular endothelial growth factor or osteoclast hyperactivity. It occurs more often in children and can involve any bone. Current therapies include surgery, radiotherapy, and pharmaceuticals [3], but there is no consensus about treatment of this rare disease. Most recently, Tena ME et al. [4] reported two cases with GSD. One went into remission when biological reconstruction was performed on three occasions, but the other died due to spinal cord compression after bisphosphonate and radiotherapy treatments. In past years, sirolimus was used for GSD in some cases, but its effectiveness is unknown. In this report, we describe the effectiveness and adverse effects of sirolimus for GSD as the basis for evaluating its therapeutic potential for this rare disease.

We report a case of a boy with GSD involving the clavicle and femur, who presented with pleural effusion as well as femoral fractures. Considering his age and side-effects of potential therapies, we gave him the mTOR (mammalian target of rapamycin) inhibitor sirolimus (rapamycin). Sirolimus is an immunosuppressant agent that directly targets mTOR. mTOR is a serine threonine kinase regulated by phosphoinositide 3 kinase (PI3K) and protein kinase B (Akt). The PI3K-Akt -mTOR signaling pathway is closely related to cell growth and proliferation and increases the expression of vascular endothelial growth factor (VEGF), regulating angiogenesis and lymphangiogenesis. mTOR inhibitors block downstream protein synthesis and have anti-tumor and anti-angiogenic effects. Sirolimus treatment was successful and the patient recovered completely. We describe the case to publicize the effect of sirolimus and provide an additional therapy for GSD. To date, 24 cases of GSD treated with sirolimus have been reported. All of the patients had bone lesions, and most of the patients were very responsive to sirolimus.

## Case presentation

**Fig. 1:**
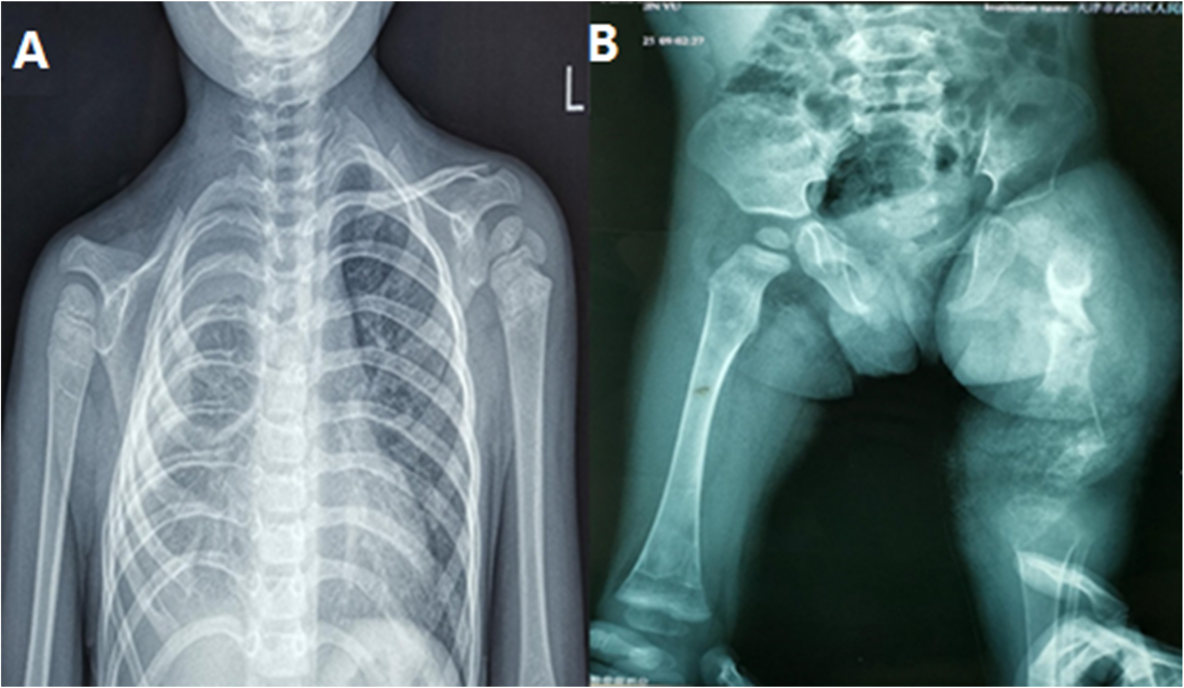
(**a**) X-ray showing osteolysis in the right clavicle. A large pleural effusion was observed on the right-side, and the left lung was significantly compressed. (**b**) X-ray showing the fracture of the left femur

**Fig. 2:**
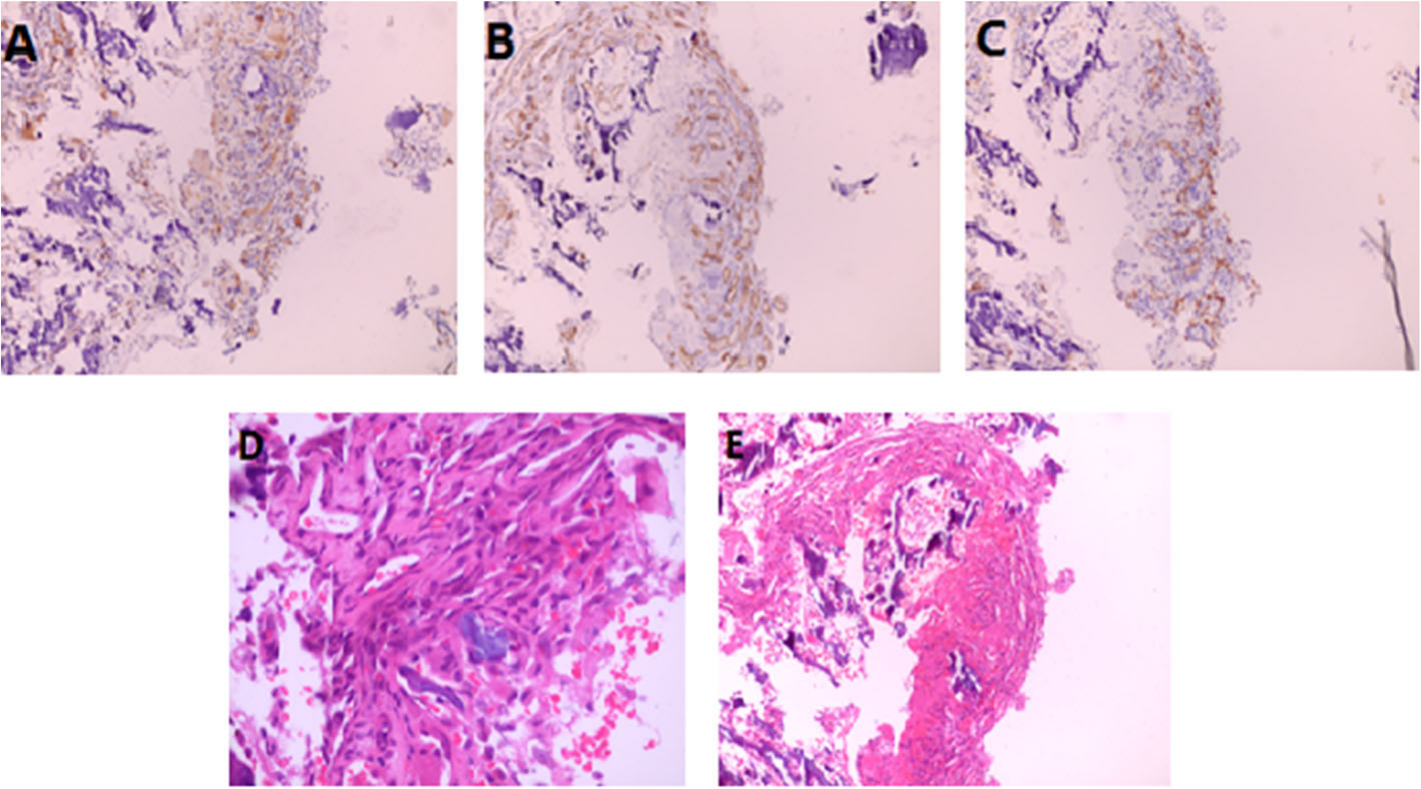
(**a**) Immunopathological examination was positive for vessel marker CD31. (**b**) Immunopathological examination was positive for vessel marker CD34. (**c**) Immunohistochemical staining for D2–40, a specific lymphatic marker, was positive in the bone tissue biopsy. (**d**) Vessel and lymphatic proliferation within necrotic bone tissue (hematoxylin-eosin [HE], 100× magnification). (**e**) Vessel and lymphatic proliferation within necrotic bone tissue (hematoxylin-eosin [HE], 400× magnification)

A 1-year-old boy was born with a 2 cm × 1.5 cm mass on the right side of his neck. He was admitted to a local hospital for treatment, and the neck CT showed cystic lesions on the superior and inferior aspects of the right clavicle, extending to the mediastinum. The local doctors gave a clinical diagnosis of suspected-lymphangioma according to radiological and clinical information. However, treatment was not administered at that time. Over time, however, he presented shortness of breath owing to a right pleural effusion with the mass gradually enlarging to 4 cm × 5 cm and came to the hospital. Physical examination showed that the mass was soft without specific boundaries, the skin was not red or swollen and there was no ulceration or fluid leakage. The chest wall behind the right clavicle collapsed with the clavicle absent causing the shoulder to droop. Respiratory sounds on the right side were faint whereas the left side was normal. The left femur was swollen and tender. The mobility of the right arm was slightly limited, and the patient found it difficult to walk. There was no significant shortness of the femur, and the arch mobility was normal. Furthermore, an X-ray showed partial osteolysis of the right clavicle with fracture of the left femur. X-rays also showed right pleural effusion with atelectasis on the right side (Fig. 1) A thoracoscopy was performed and the boy was given closed thoracic drainage via a pleural tube. After this surgery, the tumor shrank significantly. Following this, a femoral biopsy was performed that showed necrotic tissue in the cortical bone and many irregularly shaped capillaries that proliferated within the necrotic tissue. Dilated lymphatic vessels were seen adjacent to the cortex, with fibrous tissue hyperplasia (Fig. 2).

**Fig. 3:**
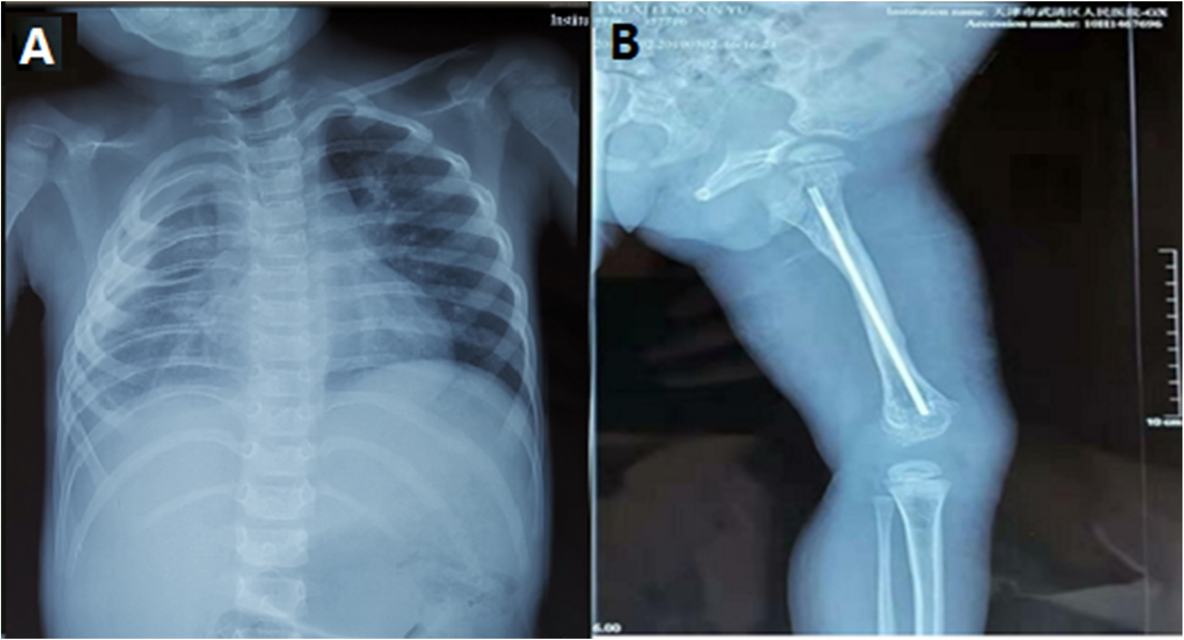
(**a**) X-ray showing a little pleural effusion in the right thoracic cavity. (**b**) X-ray of the left femur showing that the bone is remodelling well

Based on these observations, the boy was diagnosed with Gorham Stout syndrome in November 2016. The boy was given sirolimus therapy (Hangzhou Sino-American East China Pharmaceutical Co. Ltd., 1 mg/ml) simultaneously with SMZ (Shandong Xinhua Pharmaceutical Co. Ltd., 400 mg Sulfamethoxazole & 80 mg Trimethoprim /piece) at an oral dose of 20-30 mg/kg. Additionally, the patient received intramedullary fixation with bone grafting in the left femur. The dose of SMZ was divided in half and given twice a day, 3 days per week to prevent pneumocystis infection. Twenty-six months later, the boy had responded well to treatment. Physical examination showed that the mass had disappeared completely. Although the chest wall behind the right clavicle and shoulder were the same, respiratory sounds on the right side were more distinct than before. The left femur returned to nearly normal. X-rays showed that the pleural effusion was reduced significantly, there were no further lesions in the femur, and the thigh was no longer swollen (Fig. 3).

**Fig. 4:**
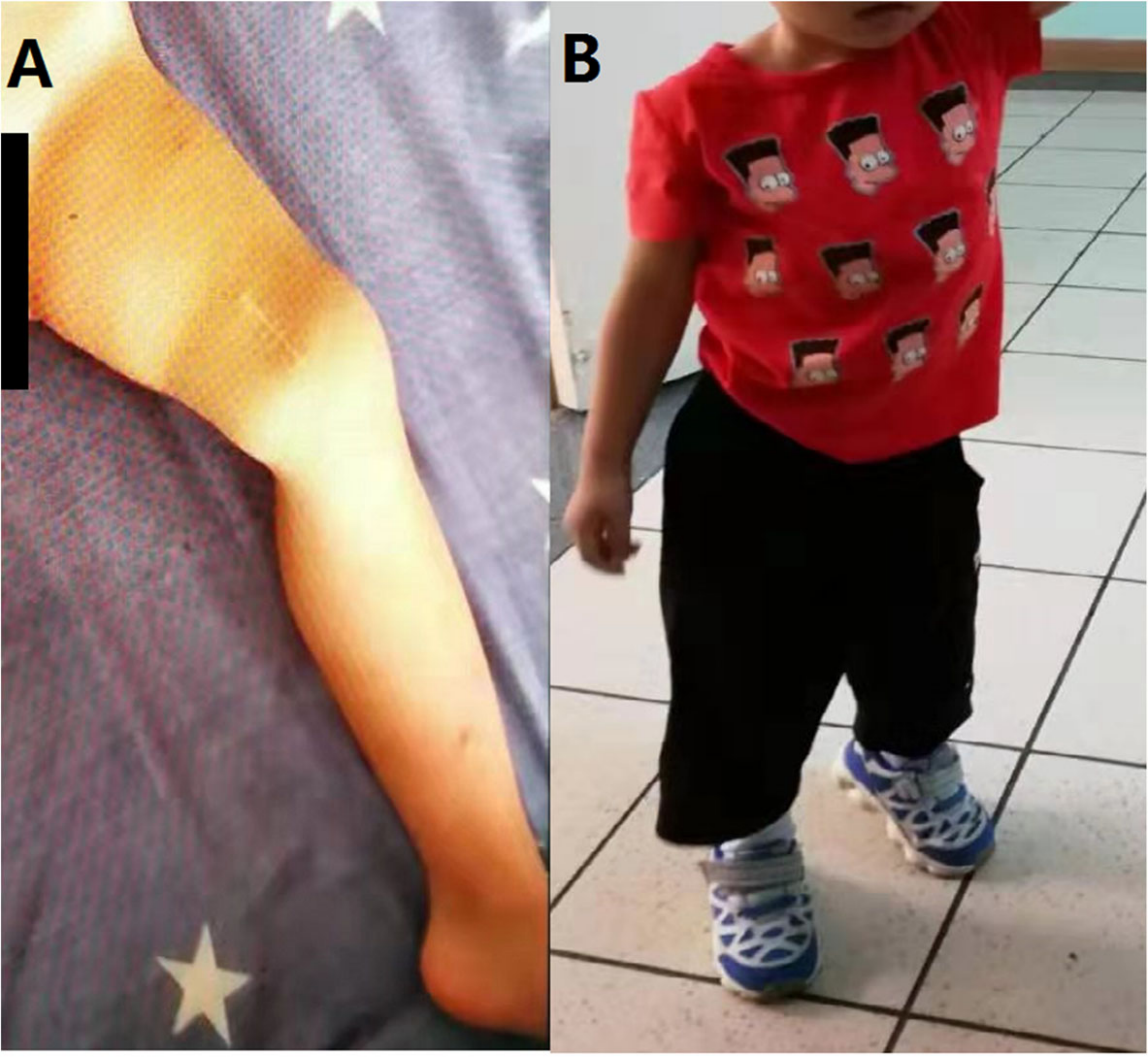
(**a**) The left femur healed without deformity and swelling was absent. (**b**) The boy can walk normally and can perform activities of daily living without restriction

Fortunately, the boy did not experience any complications while taking sirolimus. At present he has recovered well and participated in normal daily activities. His growth and development are the same as that of his peers, and he has been minimally affected by the disease (Fig. 4) (figures were published authorized by the patient’s family).

## Discussion and conclusion

**Fig. 5:**
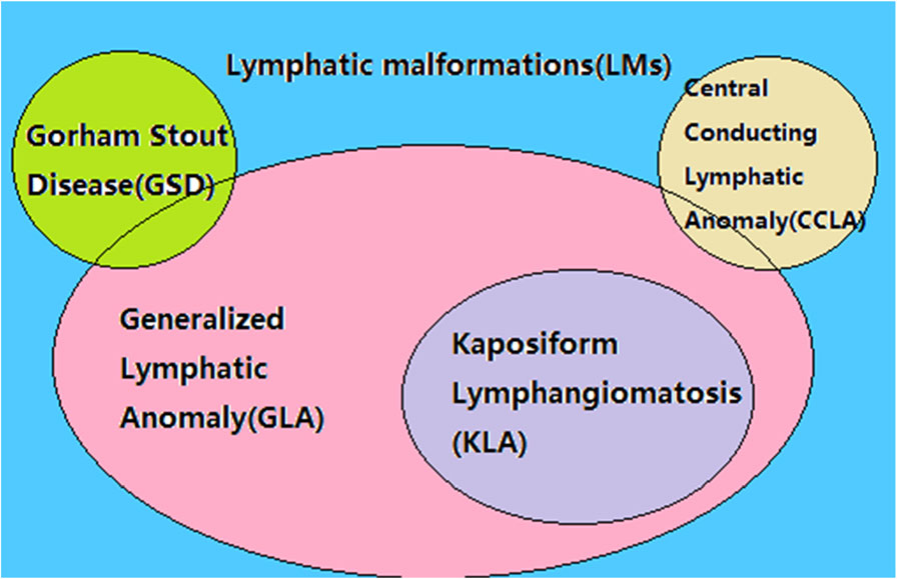
ISSVA classification of lymphatic malformations. Complex lymphatic anomalies overlap their clinical symptoms and characteristics

Lymphatic malformations (LMs) are benign vascular lesions caused by embryological abnormalities in the development of the lymphatic system [18]. In 2014, the International Society for the Study of Vascular Anomalies (ISSVA) updated the classification, which was approved at the 20th workshop (Fig. 5). Because clinical findings overlap, and disease etiology management, outcomes, and sequelae are unclear, intractable LMs (termed complex lymphatic anomalies, CLAs) are difficult to diagnose. Interestingly, osteolytic lesions are useful for distinguishing between general lymphatic anomalies (GLA)/Kaposiform lymphatic anomalies (KLA) and GSD. GSD is characterized by cortical resorption and progressive, often extensive, osteolysis, accompanied by adjacent soft tissue changes. In contrast, GLA involves organs such as lung, spleen and diffuse proliferating lymphatic vessels, with osteolysis confined to the medullary cavity and no cortical destruction. Particularly, patients with KLA have significant coagulation dysfunction with hemorrhagic pericardial and pleural effusion. The histological hallmark of KLA is a cluster of Kaposiform, spindle-shaped lymphatic endothelial cells and accumulation of hemosiderin [19].

Gorham–Stout Disease (GSD) is a rare disorder characterized by progressive osteolysis and proliferation of lymphatic and blood vessels [20]. The clinical presentation commonly involves pain and bone fractures. While the mechanisms of osteolysis in GSD remain unclear, researchers have proposed a variety of hypotheses. As it is known that GSD osteolysis occurs adjacent to local proliferation of lymphatic vessels, the activation of osteoclasts and lymphangiogenesis is essential for the progress of GSD [21]; lymph is frequently seen in the osteolytic lesions. Franco-Barrera et al. [22] reported that the activation of the osteoclastogenic process that affects bone tissue is induced by immune mechanisms.

Due to the low incidence of GSD, current literature is confined to case reports and case series. To date, this is the largest analysis of the use of sirolimus therapy in patients with GSD. Sirolimus appears to stabilize or reduce signs/symptoms of disease and improve quality of life in these affected individuals. In 2011, an early report showed sirolimus was used for GSD with good results. A total of 16 patients including the present case and conference abstracts with detailed histories have been reported in the peer-reviewed literature (Table 1). In this report, patients were 1 to 43 years old, but 62.5% of them were children under the age of 14. There was no sex difference in incidence, with 8 males and 8 females, consistent with previous reports of GSD cases. The clinical manifestations varied. Among them, nine patients had pleural effusion or chylothorax. These patients were subjected to more than one therapy, e.g., surgery or medication. Before administering sirolimus, zoledronic acid, interferon, or propranolol were given, indicating that the current therapeutic protocols are not uniform (Table 2). In addition to cases listed in Table 1, Ricci et al. [23] and Ozeki et al. [24] reported eight cases treated with sirolimus but did not provide clinical histories of these cases. Six of the eight cases responded well to sirolimus. Therefore, the overall positive response rate was 83.3% (20/24) of cases. Only one patient developed adverse effects of restrictive lung disease. He was given supportive care after stopping sirolimus treatment. These results show that sirolimus was well tolerated, even in neonates, without significant adverse effects.

**Table 1:** Reported cases of GSD treated with sirolimus (rapamycin)

Author	Gender	Age^**a**^	Nationality	Therapy	Sirolimus Dose	Manifestation	Follow up after treatment	Outcome
Cramer et al. [5]	male	18 yrs	U.S	Sirolimus zoledronic acid thoracoscopic surgery	Titrated to 9-12 mg/L, twice daily	intractable pleural effusion, and multiple rib fractures	18 mos	effusion improved and no further skeletal fracture
Araujo et al. [6]	male	5 yrs	Brazil	Sirolimus Interferon	1 mg/twice daily	ribs and sternum lesions and haemothorax	8 mos	pulmonary parenchyma normal and good bone remodelling
Garcia et al. [7]	female	43 yrs	Spain	Sirolimus Surgery	Titrated to 4-10 mg/L, twice daily	pleural effusion, abdominal mass, rib	2 mos	symptoms vanish
Mohammad et al. [8]	male	12 yrs	Malaysia	Sirolimus	NA	right chylothorax, right clavicle, humerus and ulna osteolysis	NA	responded well
Mohammad et al. [8]	male	8 yrs	Malaysia	Sirolimus	NA	cervical and thoracic spine lytic bone lesion and massive left pleural effusion	3 mos	responded poorly
Colin et al. [9]	female	3 yrs	Mexico	Sirolimus	0.05 mg/kg/d	right femur, skull osteolysis, left femur pathologic fracture	NA	control of the disease with no pain
Cakir et al. [10]	female	2 yrs	Turkey	Sirolimus	NA	right sided pleural effusion, ribs and thoracic and lumbar vertebral lytic lesions	14 mos	symptoms and signs vanish
Wei et al. [11]	female	17 yrs	U.S	Sirolimus	NA	right femur pathological fracture	7 mos	symptoms and signs vanish
Hall et al. [12]	female	10 yrs	U.S	Sirolimus	NA	right 9th rib osteolysis	24 mos	controlled
Gordon et al. [13]	female	13 yrs	Britain	Sirolimus	Titrated to 10-15 mg/L, twice daily	multiple vertebral body and pelvic osteolysis	NA	chylous leakage ceased, pain remission, and no further bone destruction
Mo et al. [14]	male	14 yrs	U.S	Sirolimus	Titrated to 7–13 mg/L, twice daily	thoracic spine and rib osteolysis	26 mos	symptoms and signs vanish
Wang et al. [3]	female	3 yrs	China	Sirolimus	Titrated to 10-15 mg/L, twice daily	humerus and scapula osteolysis and resorption in clavicle. right-sided pleural effusion	13 mos	The mass shrank and discolored skin lessened
Nozawa et al. [15]	male	27 yrs	Japan	Sirolimus+ radiotherapy+ bisphosphonates	2 mg/day	extraosseous soft-tissue mass; multiple osteolysis	6 mos	responded poorly
Suero et al. [16]	male	26 yrs	Germany	Sirolimus+ thoracoscopic pleurodesis	NA	compression fracture of the vertebrae and pleural effusion	48 mos	Recurrence (30 months after withdrawal)
Cho et al. [17]	female	11 yrs	Korea	Sirolimus+ propranolol	Titrated to 9-12 mg/L, twice daily	right clavicle and 1st rib osteolytic cortical resorption and chyle leakage	24 mos	symptoms are well-controlled
Present case	male	1 yr	China	Sirolimus+ thoracoscopic surgery	Titrated to 7–13 mg/L, twice daily	clavicle resorption. right-sided pleural effusion and right femur fracture	26 mos	symptoms and signs vanish

^a^ Age at initiation of sirolimus treatment

**Table 2:** Characteristics of reported cases^a^

Characteristics	Present patient (Y=Yes N=No)	Reports (number)
Symptoms and signs		
Osteolytic lesions	Y	15
Chylothorax	N	3
Abdominal mass	N	1
Pleural effusion	Y	5
Gender		
Male	Y	7
Female	N	8
Medical Therapy		
Bisphosphonate	N	2
Propranolol	Y	3
Sirolimus	Y	15
Surgery		
Thorascopic	Y	2

^a^ Based on the 16 studies in Table 1

The boy in this case was admitted to the hospital with a mass on the right side of his neck and neck pain. X-rays indicated partial osteolysis of the right clavicle. Normal bone tissue was replaced by proliferating non-neoplastic vascular tissue, like a hemangioma or lymphangioma. With the development of the disease the boy sustained a femoral fracture and developed pleural effusion. The diagnosis of GSD is extremely difficult, particularly in the early stage. Therefore, the diagnosis of GSD often is based on exclusion criteria (considering age, manifestation, radiology) and on the analysis of a bone biopsy. The understanding of molecular and cellular mechanisms leading to progressive osteolysis is inadequate.

Recently, some serum biomarkers have been highlighted as potentially diagnostic tools for GSD [20]. These include telopeptide of type I collagen (ICTP), Sclerostin, VEGF-A and IL-6. The sensitivity and specificity of these markers requires more clinical trials to verify their diagnostic utility. There is no standardized therapy for GSD. If surgery is not possible, radiotherapy or medication are alternative treatment modalities. Recently, several researchers have reported the mTOR inhibitor sirolimus can prevent lymphangiogenesis and decrease lymphatic endothelial cell activity [25] without adverse effects on normal lymphatics [26]. Because of the absence of evidence-based treatment guidelines, the dose and duration of sirolimus used in different studies varies significantly. In most studies, the initial dose was 0.8 mg/m^2^ of body surface area at 12-h intervals, or twice daily, and the targeting concentration in blood was 5–15 ng/ml or 10–15 ng/ml [27]. The disease went into complete remission with decreased pleural effusion, reduced lymphatic leakage and the arrest of osteolytic progression. This may offer an additional therapy that Chinese physicians can use. However, the sample size is small, so further studies are needed to confirm the efficacy of sirolimus for GSD.

The patient had the disease, with involvement of the left clavicle and the right femur, when he was born. Later he developed right pleural effusion and dyspnea. Thoracoscopy and closed thoracic drainage were performed. At that point, a fracture of the left femur was identified, and the biopsy results suggested a diagnosis of GSD. The image of profound osteolysis of the left femur with near-complete resorption of the clavicle complicated by a right-sided pleural effusion, as well as pathology of the femur, indicated that the boy had Gorham-Stout disease. This is the second GSD case treated with sirolimus reported in China. It is also the first international review of the literature on sirolimus in the treatment of GSD and may provide guidance for the future application of sirolimus in the treatment of this disease.

GSD is a disease with an unclear pathogenetic mechanism and different potential clinical therapies. This report summarizes the clinical history, diagnosis and treatment of GSD in a patient, and reviews the recent literature on sirolimus as a treatment for GSD. In contrast to zoledronic acid and interferon, sirolimus is an oral preparation, which is convenient, improves compliance, and has fewer adverse effects. Because GSD usually occurs in children, radiotherapy can have a negative impact on the child’s growth and development, so it should not be recommended as a first line treatment. Because of its safety and efficacy, fewer adverse effects, and convenience, sirolimus may become an alternative medication for the treatment of GSD, but a larger randomized controlled trial needs to be performed.

## Data Availability

All data generated or analysed during this study are included in this published article.

## Abbreviations

GSD: Gorham-Stout disease
LM: Lymphatic anomaly
mTOR: Mammalian target of rapamycin
PI3K: Phosphoinositide 3 kinase
Akt/PKB: Protein kinase B
VEGF: Vascular endothelial growth factor
CT: Computed tomography
ISSVA: International Society for the Study of Vascular Anomalies
CLAs: Complex lymphatic anomalies
GLA: General lymphatic anomalies
KLA: Kaposiform lymphatic anomalies
ICTP: Telopeptide of type I collagen
